# Different Trichoscopic Features of Tinea Capitis and Alopecia Areata in Pediatric Patients

**DOI:** 10.1155/2014/848763

**Published:** 2014-06-16

**Authors:** Abd-Elaziz El-Taweel, Fatma El-Esawy, Osama Abdel-Salam

**Affiliations:** Dermatology & Andrology Department, Faculty of Medicine, Benha University, Benha, Al Qalyubia 13512, Egypt

## Abstract

*Background*. Diagnosis of patchy hair loss in pediatric patients is often a matter of considerable debate among dermatologists. Trichoscopy is a rapid and noninvasive tool to detect more details of patchy hair loss. Like clinical dermatology, trichoscopy works parallel to the skin surface and perpendicular to the histological plane; like the histopathology, it thus allows the viewing of structures not discovered by the naked eye. *Objective*. Aiming to compare the different trichoscopic features of tinea capitis and alopecia areata in pediatric patients. *Patients and Methods*. This study included 40 patients, 20 patients with tinea capitis and 20 patients with alopecia areata. They were exposed toclinical examination, laboratory investigations (10% KOH and fungal culture), and trichoscope examination. *Results*. Our obtained results reported that, in tinea capitis patients, comma shaped hairs, corkscrew hairs, and zigzag shaped hairs are the diagnostic trichoscopic features of tinea capitis. While in alopecia areata patients, the most trichoscopic specific features were yellow dots, exclamation mark, and short vellus hairs. *Conclusion*. Trichoscopy can be used as a noninvasive tool for rapid diagnosis of tinea capitis and alopecia areata in pediatric patients.

## 1. Introduction

Losing hair is not usually health threatening; it can scar a young child's vulnerable self-esteem by causing immense psychological and emotional stress, not only to the patient, but also to the concerned parents and siblings [1]; so the cause of hair loss should be diagnosed and treated early to overcome the resulting problems [2]. The most frequent causes of hair loss in pediatric patients include tinea capitis, alopecia areata, traction alopecia, and trichotillomania. The clinician must be able to separate the types and causes of hair loss into those that reflect primary dermatologic conditions and those that represent reaction to systemic disease [3].

Tinea capitis is a superficial fungal infection of the scalp. The disease is primarily caused by dermatophytes in the* Trichophyton* and* Microsporum* genera that invade the hair shaft. The clinical presentation is typically a single or multiple patches of hair loss, sometimes with a “black dot” pattern, that may be accompanied by inflammation, scaling, pustules, and itching [4]. Alopecia areata (AA) is a medical condition in which hair is lost from some or all areas of the body, usually from the scalp [5]. Typical first symptoms of alopecia areata are small bald patches; the underlying skin looks superficially normal. These patches can take many shapes but are most usually round or oval [6].

The cause of focal hair loss may be diagnosed by the appearance of the patch and examination for fungal agents. A scalp biopsy may be necessary if the cause of hair loss is unclear [7].

Trichoscopy is a noninvasive diagnostic tool that allows the recognition of morphologic structures not visible by the naked eye. The trichoscope, despite its ease of handling, is not a mere magnifying glass but a more complex instrument, allowing the superimposition of the skin layers. This is entirely different from the image obtained in histopathology, where the visualization is total, with the possibility to observe any surface or deep skin layer [8]. Trichoscopy is useful for the diagnosis and follow-up of hair and scalp disorders. However, it is not widely used in the management of hair disorders. This will enable dermatologists to make fast diagnoses of tinea capitis and alopecia areata, distinguish early androgenetic alopecia from telogen effluvium, and differentiate scarring from nonscarring alopecia [9].

*Aim of Work.* The aim of this work is to detect different trichoscopic features of tinea capitis and alopecia areata in pediatric patients with localized patches of hair loss.

## 2. Patients and Methods 

This observational analytical study included forty patients, twenty patients with tinea capitis and twenty patients with alopecia areata, without sex predilection, their age (≤12 years) presented with solitary or multiple lesions of patchy hair loss of the scalp. This study was conducted at Cairo Hospital of Dermatology and Andrology (Al-Haud Al-Marsoud), during the period from October 2012 to March 2013.* The exclusion criteria* were (1) patients with any concomitant dermatological diseases and (2) history of using any topical (1 month) or systemic treatment (3 month) for tinea capitis or alopecia areata prior to the study.* Consent* was taken from a parent of each patient before participation in the study which was approved by Ethics Committee Human Research of Benha University.

All patients were subjected to the following: (1) history taking, clinical examination, and digital photography of any lesion of patchy hair loss by using Panasonic LUMIX S5 16 mega pixel, (2) microscopic examination of skin scraping and plucked hairs using KOH 10% and fungal culture and (3) trichoscopic examination.

### 2.1. Laboratory Examination

The specimen was collected in a sufficient amount from the edge of the area of hair loss (scales or plucked hairs). Hair roots and skin scraping were mounted in 10% potassium hydroxide solution. The slide was gently heated and microscopically examined for spores. The culture was done on Sabouraud's agar media; the cultures were incubated at 30°C and examined frequently for 4 weeks.

### 2.2. Trichoscope Examination

In this study, a hand-held trichoscope (DermLite DL3, Gen, USA) which can block light reflection from the skin surface without immersion gels was used. Characteristics and specifications of the DermLite DL3 trichoscope used in the study were 20x and 40x magnification with focusing optics. It consisted from light emitting diodes (LEDs) bulbs, piece handle and head, and replaceable batteries (rechargeable lithium).

The trichoscope is switched on and placed away from the lesion by about 1 cm or gently over the lesion after covering the lesion with gel, so that it is in the center of the contact plate. The examiner's eyes should be as close as possible to the eyepieces, with the free hand adjusting the focusing ring until a clearly focused image is obtained (in most cases it is only necessary to set up the focus once). Disinfection of the lens with alcohol swab to avoid transmission of infection. Digital photography of the lesion(s) were taken through the DermLite DL3 Gen Trichoscope. Findings obtained were evaluated by the same two dermatologists.

### 2.3. Statistical Analysis

All collected data were revised for completeness and accuracy. Precoded data was entered on the computer using the statistical package of social science software program, version 15 (SPSS), to be statistically analyzed.

## 3. Results 

### 3.1. Clinical Data

In alopecia areata patients, the study was carried on 13 female 65% and 7 males 35%. Their age ranged from 1.5–11 years with median ± inter quartile range (IQR) 5.25 (3.3, 8.0). The duration of lesions ranged from 2 to 12 weeks with median ± IQR 4.00 (2.3, 11.0). The number of lesion(s) ranged from 1 to 2 with median ± IQR 1.0 (1.0, 2.0). The size of the lesion(s) ranged from 0.5 to 3 cm with median ± IQR (2.0 ± 0.6 × 1.5 ± 0.7). In tinea capitis patients, the study was carried on 15 male 75.0% and 5 females 15.0%. Their age ranged from 2–11 years with median ± IQR 5.0 (3.5, 7.1), the duration of the lesion(s) ranged from 2 to 12 weeks with median ± IQR 4.00 (2.3, 11.0). The number of lesion(s) ranged from 1 to 2 with median ± IQR 1.0 (1.0, 1.0). The size of the lesion(s) ranged from 1 to 3 cm with median ± IQR (2.1 ± 0.8 × 1.6 ± 0.8).

### 3.2. Laboratory Results

Direct microscopic examination of the collected specimens from the lesion(s) after being mounted by KOH 10% was done for all patients and revealed that 13 patients, 32.5%, of tinea capitis gave positive result, 7 patients, 17.5%, gave false negative results, and all cases of alopecia areata gave negative results. The dermatophytes isolated are as follows:* T. violaceum* in 6 patients, 15.0%,* M. canis* in 6 patients, 15.0%,* T. rubrum* in 3 patients, 7.0%, and* T. verrucosum* in 5 patients, 13.0%.

### 3.3. Trichoscopic Results

**Figure 1:**
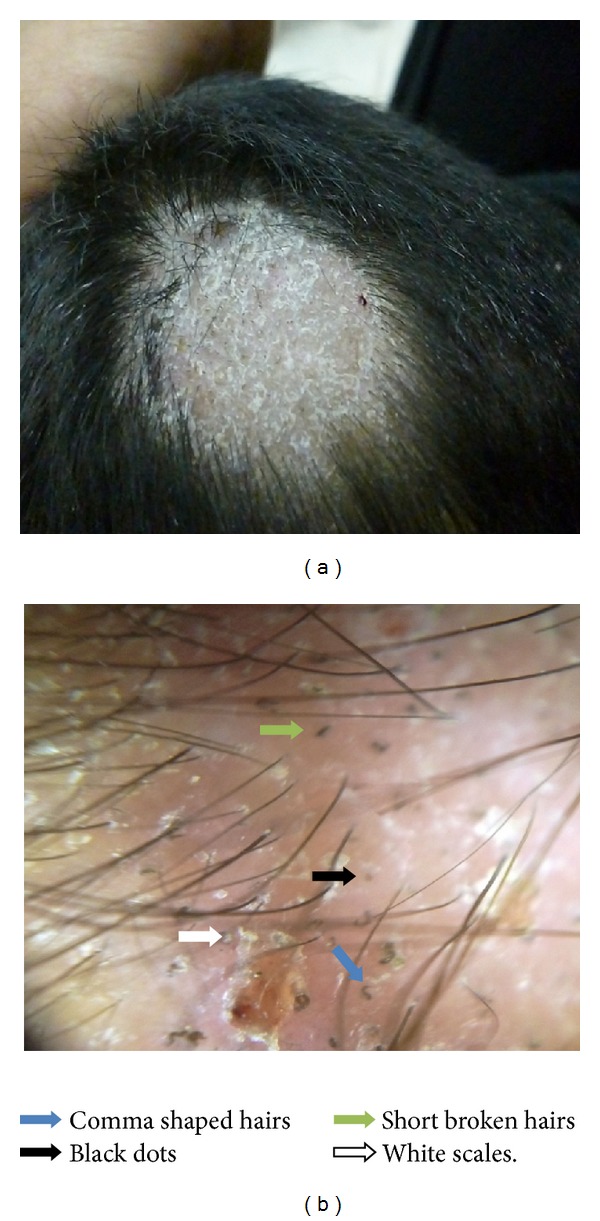
Tinea capitis (a) macroscopic view, (b) trichoscopic view at 20x magnification, dermoscopy shows comma shaped hairs (blue arrow), black dot (black arrow), short broken hairs (green arrow), and white scales (white arrow).

In patients with tinea capitis, the most common trichoscopic feature (Figure 1) was short broken hairs seen in 18 patients, 90.0%, followed by black dots in 13 patients, 65.0%, comma shaped hairs in 11 patients, 55.0%, or corkscrew hairs in 9 patients, 45.0%, and zigzag shaped hair in 5 patients, 25.0% (Table 1).

**Figure 2:**
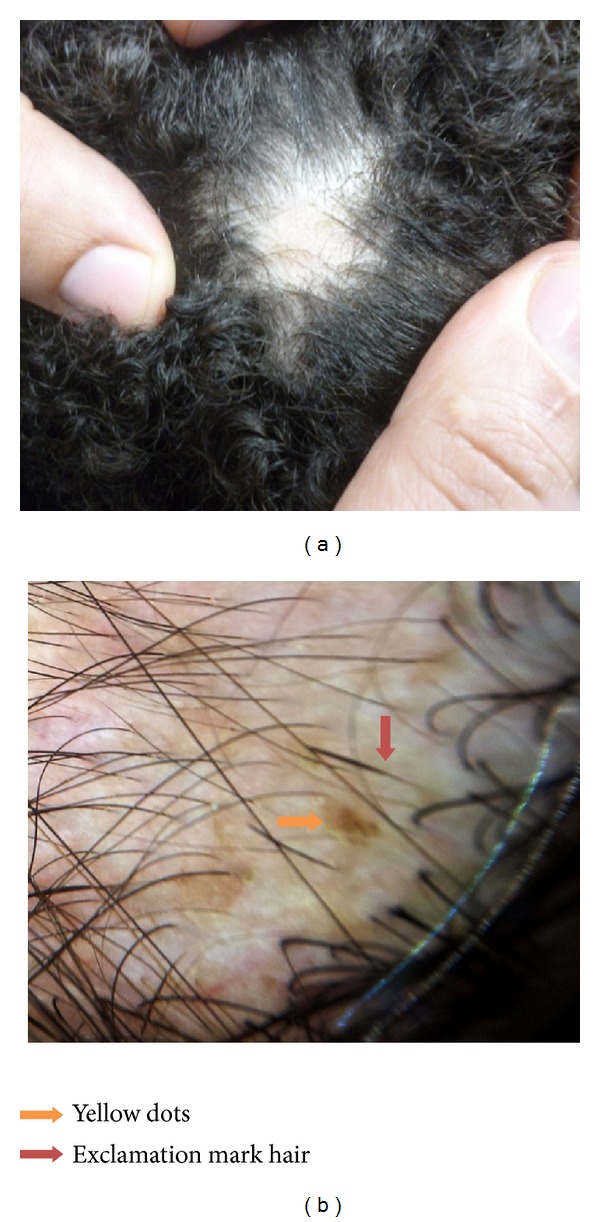
Alopecia areata (a) macroscopic view, (b) trichoscopic view at 40x magnification, shows yellow dots (orange arrow) and exclamation mark hair (pink arrow).

In patients with alopecia areata the most common trichoscopic feature (Figure 2) was black dots in 12 patients, 60.0%, followed by yellow dots seen in 11 patients, 55.0%, exclamation mark in 11 patients, 55.0%, white hairs in 9 patients, 45.0%, short vellus hairs in 8 patients, 40.0%, short broken hairs in 8 patients, 40.0%, and pig tail growing hairs in 3 patients, 15.0% (Table 2).

## 4. Discussion

Tinea capitis and alopecia areata are considered to be the most common causes of hairless patches of the scalp in pediatrics [10]. Tinea capitis especially nonscaly type may have the same clinical appearance of alopecia areata, so trichoscopy has recently become a useful diagnostic tool for alopecia areata and tinea capitis, especially in doubtful cases as lab investigations like fungal culture or biopsy may take several weeks [11, 12].

The studies regarding trichoscopic finding of patients with tinea capitis were few and included few patients [13]. In the present study we found in tinea capitis patients by trichoscope examination, comma shaped hairs, zigzag shaped hairs, corkscrew hairs, black dots, and shortbroken hairs are considered characteristic trichoscopic features of tinea capitis as conducted by Ekiz et al. [14].

In the present study, comma shaped hairs were seen in 55% (11 out of 20 patients); this finding was detected in other studies that included few number of patients [14–16]. Comma hairs, which are slightly curved and fractured hair shafts, are associated with ectothrix and endothrix type fungal invasion. The authors believe that comma hair is probably shaped as a result of subsequent cracking and bending of a hair shaft filled with hyphae [15].

In the current study, zigzag shaped hairs were seen in 25.0% (5 out of 20 patients) and corkscrew hairs in 45.0% (9 out of 20 patients); these findings were detected in other studies with different number of patients [14, 16]. The zigzag shaped hairs or corkscrew hair seems to be a variation of the comma hair, manifesting in black patients [16].

Short broken hairs were observed in the present study in 90.0% (18 out of 20 patients) of tinea capitis cases; this finding was conducted with other studies [13, 14]. Short broken hairs may be nonspecific trichoscopic finding of tinea capitis but may be a sign of severity of the disease.

Black dots were reported in our study in 65.0% (13 out of 20 patients) of tinea capitis cases, as conducted by Sandoval et al. [17]. Black dots are remnants of broken hairs or dystrophic hairs [18].

Hughes et al. [16] stated that comma shaped hairs, corkscrew hairs were detected in zoophilic infection. In the present study* T. violaceum*,* M. canis*, and* T. verrucosum* were isolated; this result may be due to farming and low socioeconomic status of our patients.

To conclude the most common trichoscopic features are short broken hairs, followed by black dots, comma shaped hairs, or corkscrew hairs. However, comma shaped hairs, zigzag shaped hairs, or corkscrew hairs are characteristic trichoscopic features of tinea capitis. Black dots and short broken or dystrophic hairs are not specific to tinea capitis, as they can be observed also in alopecic areata and trichotillomania but could be used as a sign of severity of tinea capitis.

There are large scale studies in patients with alopecia areata. Yellow dots, black dots, broken hairs, exclamation mark, and short vellus hairs are considered as characteristic trichoscopic features in AA [14, 19].

In the present study yellow dots are detected in 55% (11 out of 20 patients) of alopecia areata patients; this finding was detected in other studies [20, 21]; these are marked by distinctive array of yellow to yellow-pink, round or polycyclic dots that vary in size and are uniform in color. They are more easily observed using video trichoscopy than with handheld trichoscopy [18]. The combination of large numbers of yellow dots and short growing hairs is a feature of AA incognita [22]. For the diagnosis of alopecia areata, other signs of alopecia areata should be taken into account, because isolated yellow dots may be seen in trichotillomania, hypotrichosis simplex, and even tinea capitis, as stated by Inui [23].

In the current study under trichoscopic examination exclamation mark hair was detected in 55.0% (11 out of 20 patients) of alopecia areata cases; this finding was detected in other studies [14, 18, 20]. This term, tapering hair, is preferred over “exclamation mark hair” because the affected hair is not typical exclamatory mark in shape. It occurs due to the narrowing of hair shafts toward the follicles which is more readily perceived using trichoscopy than by naked eye [18]. In the present study we believe that tapering hairs are diagnostic feature of alopecia areata as reported by authors [12, 14, 19]. We discovered that it was more sensitive and diagnostic when associated with yellow dots, short vellus hairs, or pig tail growing hairs. We detected that it was sign of active alopecia areata, as it was seen in the active cases of alopecia areata at the periphery of the lesion(s).

In our study under trichoscopic examination black dots were detected in 60.0% (12 out of 20 patients) of tinea capitis patients; this finding was detected in other studies [19, 20]. Black dots as remnants of exclamation mark hairs or broken hairs occur when hair shaft, fractured before emerging from the scalp, provides a sensitive marker for disease activity as well as severity of AA [18]. The present study showed that black dots are the most common trichoscopic finding and can be used as a sensitive feature of alopecia areata only if associated with other specific trichoscopic features of alopecia areata as yellow dots, tapering hairs, or short vellus hairs. As in the present study, black dots were detected also in cases of trichotillomania and tinea capitis, and black dots were not detected by Ekiz et al. [14].

In the present study under trichoscopic examination short vellus hair was detected in 40.0% (8 patients out of 20 patients) of alopecia areata cases; this finding was detected in other studies [14, 19, 20]. Short vellus hairs were seen as new, thin, and nonpigmented hairs within the patch, which may or may not be clinically detectable [24]. Our obtained data showed that short vellus hair is also a diagnostic feature of AA, which can provide useful prognostic information (indicating the nondestructive nature of AA) as stated by Inui et al. [18]. They also mentioned that the appearance of clusters of short vellus hairs is a possible sign of spontaneous remission or adequate treatment, but in the present study it was a sign of spontaneous remission as the cases were not treated before the study.

In the current study pig tail regrowing hair was reported in 15.0% (3 out of 20 patients) of alopecia areata patients; this finding was detected in other studies [19]. We observed that pig tail growing hair is not common, but if present it is a diagnostic trichoscopic finding and is a possible sign of spontaneous remission of alopecia areata.

In the present study short broken hairs were detected in 40.0% (8 out of 20 patients) of alopecia areata cases conducted with other authors [18–20]. Inui et al. [18] mentioned that broken hairs were considered as being clinical markers of the disease activity and severity of AA. They are nondiagnostic as in our study we detected broken hairs in tinea capitis cases as mentioned by Köse and Güleç [19] and Ekiz et al. [14].

In the present study white hairs were detected in 45.0% (9 out of 20 patients) of alopecia areata cases; we suggested that it is a diagnostic trichoscopic finding and a sign of spontaneous remission of alopecia areata.

Some of the trichoscopic features can be used to predict the activity and severity of AA. Tapering hair is considered as a marker of disease activity and known to reflect exacerbation of disease. These trichoscopic findings will be helpful for management of patients with hair disorders. Yellow dots and short vellus hairs enable AA to be screened from other hair loss disorders. Abundant numbers of the yellow dots seen in AA can differentiate it from trichotillomania which can have limited number of yellow dots. In addition, black dots, tapering hairs, and broken hairs are specific for AA, except for trichotillomaniasingle trichoscopic feature which may not reliably diagnose AA [20]. Inui et al. [18] found that a combination of cadaverized hairs, exclamation mark hairs, broken hairs, and yellow dots could sensitively detect difficult-to-clinically diagnose types of AA like alopecia areata incognita, and broken hairs may be found in tinea capitis and trichotillomania.

To conclude the most common trichoscopic feature was black dots, followed by yellow dots, exclamation mark, white hairs, short vellus hairs, short broken hairs, and pig tail growing hairs. However, yellow dots, exclamation mark hair, and short vellus hair are specific to alopecia areata; if not detected under trichoscopy, further clinical and histopathological examination will be required.

## 5. Conclusion

Trichoscopy has been shown to improve the clinical diagnostic performance in the daily practice; it can be used to differentiate between tinea capitis by its characteristic findings as comma shaped hairs and zigzag shaped hairs or corkscrew hairs which are not present in alopecia areata. Alopecia areata also has characteristic findings as yellow dots or exclamation mark which are not present in tinea capitis. Trichoscopy can nowadays be seen as the dermatologists' stethoscope.

## Figures and Tables

**Table 1:** Different trichoscopic features of tinea capitis.

	Frequency (*N* = 20)	Percent (%)
Comma shaped hairs		
Present	11	55.0
Absent	9	45.0
Zigzag shaped hairs		
Present	5	25.0
Absent	15	75.0
Black dots		
Present	13	65.0
Absent	7	35.0
Short broken hairs		
Present	18	90.0
Absent	2	10.0
Corkscrew hairs		
Present	9	45.0
Absent	11	55.0

The most common trichoscopic feature was short broken hairs followed by black dots, but both of them are nonspecific as they were detected in other conditions of hair loss. Comma shaped hairs, corkscrew hairs, and zigzag shaped hairs are the diagnostic trichoscopic features of tinea capitis.

**Table 2:** Different trichoscopic features of alopecia areata.

	Frequency (*N* = 20)	Percent (%)
Black dots		
Present	12	60.0
Absent	8	40.0
Yellow dots		
Present	11	55.0
Absent	9	45.0
Microexclamation mark		
Present	11	55.0
Absent	9	45.0
Short vellus hairs		
Present	8	40.0
Absent	12	60.0
Pig tail regrowing hair		
Present	3	15.0
Absent	17	85.0
Short broken hairs		
Present	8	40.0
Absent	12	60.0
White hairs		
Present	9	45.0
Absent	11	55.0

In alopecia areata patients, the most common trichoscopic feature was black dots but it is nonspecific for alopecia areata, as it is found in other conditions as trichotillomania and tinea capitis. But the specific features were yellow dots, exclamation mark, and short vellus hairs.
